# Image Encryption Based on Pixel-Level Diffusion with Dynamic Filtering and DNA-Level Permutation with 3D Latin Cubes

**DOI:** 10.3390/e21030319

**Published:** 2019-03-24

**Authors:** Taiyong Li, Jiayi Shi, Xinsheng Li, Jiang Wu, Fan Pan

**Affiliations:** 1 School of Economic Information Engineering, Southwestern University of Finance and Economics, Chengdu 611130, China; 2 College of Computer Science, Sichuan University, Chengdu 610064, China; 3 College of Electronics and Information Engineering, Sichuan University, Chengdu 610064, China

**Keywords:** image encryption, dynamic filtering, DNA computing, 3D Latin cube, permutation, diffusion

## Abstract

Image encryption is one of the essential tasks in image security. In this paper, we propose a novel approach that integrates a hyperchaotic system, pixel-level Dynamic Filtering, DNA computing, and operations on 3D Latin Cubes, namely DFDLC, for image encryption. Specifically, the approach consists of five stages: (1) a newly proposed 5D hyperchaotic system with two positive Lyapunov exponents is applied to generate a pseudorandom sequence; (2) for each pixel in an image, a filtering operation with different templates called dynamic filtering is conducted to diffuse the image; (3) DNA encoding is applied to the diffused image and then the DNA-level image is transformed into several 3D DNA-level cubes; (4) Latin cube is operated on each DNA-level cube; and (5) all the DNA cubes are integrated and decoded to a 2D cipher image. Extensive experiments are conducted on public testing images, and the results show that the proposed DFDLC can achieve state-of-the-art results in terms of several evaluation criteria.

## 1. Introduction

As one of the most important information carriers, hundreds of millions of images are generated, stored, and transmitted every day. How to ensure image security has become a very hot topic of research in recent years. Image encryption is one of the most important image security methods. Encryption algorithms can be roughly classified into two categories: symmetric key (private key) and asymmetric key (public key) algorithms. The former uses the same key for both encryption and decryption, while the latter uses a key for encryption and another key for decryption. Typical private key algorithms include data encryption standard (DES), international data encryption algorithm (IDEA), advanced encryption standard (AES) and so on. Rivest-Shamir-Adleman (RSA) and Elliptic-curve cryptography (ECC) are among the very popular public key algorithms. The symmetric key algorithms are fast, efficient, but difficult to manage keys, while the asymmetric encryption algorithms are slow but have higher security [1,2]. Due to the inherent characteristics of images such as strong correlation, high redundancy and bulky data capacity, the above mentioned encryption algorithms are usually not suitable for direct applications in images. To address this issue, a variety of image encryption algorithms have been proposed in recent years [3,4,5,6].

There are many kinds of operations for the purpose of image encryption, such as shuffling, permutation, rotation, substitution, confusion, diffusion, transposition, and so on [7]. Among the operations, diffusion and permutation are very popular ones because they can achieve good results and are easy to implement. The diffusion is to change the values of the pixels in images, while the permutation aims at changing the positions of the pixels. Some practical image encryption algorithms are capable of handling diffusion and permutation jointly. Due to the characteristics of ergodicity, pseudorandomness, unpredictability, and extreme sensitivity to initial values and parameters, chaos-based image encryption has become increasingly popular in recent years. The main idea of chaos-based image encryption is to conduct diffusion and/or permutation according to the pseudorandom sequences generated from chaotic systems [8,9,10,11,12,13]. Very recently, Flores-Vergara et al. have implemented a chaotic cryptosystem on embedded systems with multiprocessors. The NIST statistical test and the security analysis have confirmed the proposed cryptosystem is very secure and robust for image encryption [14]. Wang et al. used a spatiotemporal chaotic system to generate a pseudorandom sequence, and then used the sequence to conduct permutation and diffusion simultaneously [15]. Pareek et al. employed two chaotic logistic maps and eight different types of operations to encrypt the pixels of images, and the experimental results demonstrated the proposed scheme was real-time, efficient and secure [16]. Hua et al. put forward a new 2D Logistic-Sine-coupling map that has more complex behavior, better ergodicity, and larger chaotic range than some other 2D chaotic maps, for image encryption scheme. The experiments showed that the proposed scheme had better security performance than several state-of-the-art encryption approaches [17]. Sahari and Boukemara proposed a novel 3D chaotic map by integrating the piecewise and logistic maps for color image encryption, the experimental results showed the efficiency and safety of the proposed scheme [18]. Zhou et al. proposed a novel image encryption scheme by combining quantum 3D Arnold transform and quantum XOR operations with scaled 3D Zhongtang chaotic system [19]. Low-dimensional chaotic systems have the advantages of simple forms, only a few parameters, and easy implementation. However, such properties may make it easy to estimate the orbits and the initial parameters of the low-dimensional chaotic systems and hence the security of encryption is limited.

In a dynamical system, the Lyapunov exponent (LE) is used to measure the rate of separation of infinitesimally close trajectories [20]. If a chaotic system has at least two positive LEs, the system is said to be hyperchaotic. The image encryption algorithms with hyperchaotic systems have been demonstrated more secure [2,6,21,22,23,24,25,26]. Chai et al. used a 4D memristive hyperchaotic system to encrypt 4 compound bit planes recombined from the 24-bit planes of components R, G, and B [27]. Li et al. proposed a quantum image compression-encryption approach with quantum cosine transform and a 5D hyperchaotic system, and the experiments demonstrated that the proposed compression-encryption approach outperformed some classical image encryption approaches [28]. Zhou et al. used a 5D hyperchaotic system for quantum color image encryption. Some researchers also applied 6D or 7D hyperchaotic systems to generate hyperchaotic sequences for image encryption [6,29].

Like other tasks in signal processing, image encryption can also be conducted in both spatial or transform domain [30,31,32,33,34,35]. The encryption in spatial domain is very direct, which changes the values and/or the positions of pixels. To improve the efficiency or the effectiveness of image encryption, sometime the algorithms can be conducted on higher-level data (blocks of pixels) or lower-level data (DNA-level data and bit-level data) [36,37,38]. Generally speaking, for the same processing power, the lower the data level, the more pixels will be involved in encryption. Therefore, the encryption processing lower-level data usually achieves better performance of encryption [6]. In the field of image encryption, the introduction of transform domain is for the purpose of compressing images. Typical transform methods include discrete cosine transform (DCT) [39,40,41,42,43], Fourier transform [44,45,46], and wavelet transform [47,48,49,50]. With these transform methods, the image encryption can focus on the high-energy parts of the images only and discard some low-energy (zero coefficients) parts. Then the image can be recovered by decryption and corresponding reverse transform.

Some recent progress has improved the performance of image encryption. Regarding diffusion, Hua and Zhou introduced filtering, a very popular technique in image processing, into image encryption. The authors make the filtering reversible by setting the right-bottom point of the filter to “1”, and they proposed an image encryption algorithm using block-based scrambling and image filtering (BSIF) with a fixed filter for all pixels [51]. Very recently, Hua et al. have extended image encryption with Josephus scrambling and filtering diffusion, where the filter is a 2×2 square with fixed values [52]. Li et al. used a 1×3 or 3×1 filter with dynamically variable values decided by a 7D hyperchaotic system for filtering (so-called dynamic filtering), and bit cuboid operations, namely DFBC, for image encryption, and the experiments demonstrated the DFBC could achieve state-of-the-art results [6]. As far as permutation is concerned, in theory, any reversible position transform can be used for image encryption. Latin squares are such popular transforms which help to achieve good results of permutation [53,54,55]. Xu et al. extended the use of Latin squares in image encryption, and they treated the pixel-level image as a 3D bit matrix and then conducted operations of Latin cubes on the 3D matrix, and the experimental results showed that the proposed image encryption achieves both a desirable level of security and high efficiency [56].

Motivated by the diffusion with filtering and the permutation with Latin cubes, in this paper, we propose a novel approach that integratings a hyperchaotic system, Dynamic Filtering, DNA computing, and Latin Cubes, termed as DFDLC, for image encryption. Specifically, the DFDLC consists of five stages: (1) A 5D hyperchaotic system with 2 positive LEs is applied to generate the chaotic sequences for subsequent diffusion and permutation. (2) Filters with variable values are generated from the chaotic sequences, and filtering is conducted on each pixel of the image with a different filter. That is to say, the value of each pixel is changed by a different filter. This is called pixel-level diffusion with dynamic filtering. (3) The 2D pixel plane is transformed into several DNA cubes via DNA encoding rules determined by the chaotic sequence. (4) For each DNA cube, we generate a Latin cube with the same size and then change the position of each element in the DNA cube via the Latin cube. This operation is called DNA-level permutation with 3D Latin cubes. (5) All the DNA cubes are integrated and decoded to a 2D pixel image. The main contributions of this paper are three-aspect: (1) We propose a novel image encryption using a newly found 5D hyperchaotic system. (2) Pixel-level dynamic filtering and DNA-level permutation with Latin cubes are used to improve the performance of image encryption. (3) Extensive experiments on several public images show that the DFDLC is promising for image encryption.

The remainder of this paper is structured as the following. A brief description of a 5D hyperchaotic system with two positive LEs, filtering, DNA computing and Latin square is given in Section 2. In Section 3, a novel image encryption algorithm with dynamic filtering and Latin cube transformation, namely DFDLC, is proposed in detail. Experimental results are reported and analyzed in Section 4. Finally, the paper is concluded in Section 5.

## 2. Preliminaries

### 2.1. Hyperchaotic Systems

**Figure 1:**
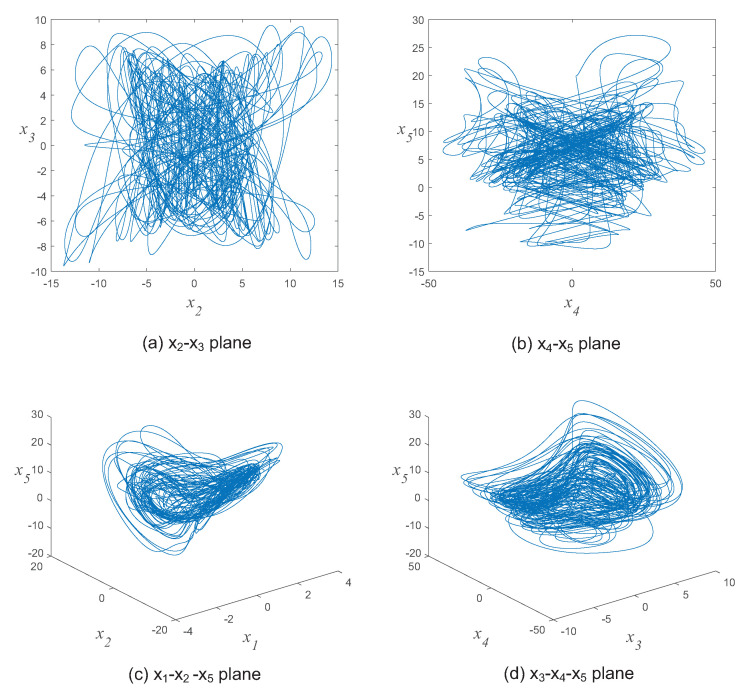
The attractors of the 5D hyperchaotic system.

As one of the most popular chaotic systems, the Lorenz chaotic system and its extensions are very popular in image encryption. Most recently, Wang et al. have found a new 5D autonomous hyperchaotic system with 2 positive LEs by adding feedback controllers to the Lorenz system, formulated as Equation (1) [57]:

(1) $$\left\{\begin{array}{c} \dot{x_{1}}=x_{2}\\ \dot{x_{2}}=-x_{2}+ax_{1}+bx_{1}^{3}+cx_{1}x_{5}\\ \dot{x_{3}}=x_{4}\\ \dot{x_{4}}=-x_{4}+dx_{3}+ex_{3}^{3}+fx_{3}x_{5}\\ \dot{x_{5}}=-gx_{5}+hx_{1}^{2}+ix_{3}^{2} \end{array}\right.$$

where $x_{j}\left(j=1,2,\cdots ,5\right)$ are state variables, and (a,b,c,d,e,f,g,h,i) are constant parameters. There are several numerical methods to solve this system, such as Forward Euler (FE), 4th order Runge-Kutta (RK) and newly proposed trigonometric polynomials [58]. In this paper, we use the 4th order RK method with a step size of h=0.001 to solve the hyperchaotic system. When the parameters (a,b,c,d,e,f,g,h,i)=(4,−1,−1,2,−1,2,0.0,6,−1) and initial values ($x_{1}^{0},x_{2}^{0},x_{3}^{0},x_{4}^{0},x_{5}^{0})=\left(1.618,3.14,2.718,4.6692,0.618\right)\times 10^{-2}$, the attractors of the 5D hyperchaotic system are shown in Figure 1.

### 2.2. Filtering

Filtering, also termed as convolution, is a very popular operation in the field of image processing, which can be applied to denoising, smoothing, and sharpening images by changing the values of pixels. Typically, the operation of filtering is to do convolution between a mask, also known as a kernel/filter/window, and an image. The values of pixels in an image are changed and hence it seems that filtering can be used for diffusion directly. However, since traditional filtering cannot be reversible, the cipher image with such diffusion cannot be recovered. To cope with this issue, Hua and Zhou set the right-bottom point of the filter to “1” and then align this point to the processed pixel in the image for convolution, and they proposed a novel image encryption algorithm with block-based scrambling and such image filtering (BSIF) [51]. However, the BSIF used a fixed filter for all pixel when doing convolution, limiting the encryption performance. An ideal scheme should use a variable/dynamic filter for convolution with each pixel.

### 2.3. DNA Computing

DNA computing, invented by Leonard Adleman, is a type of parallel computing technique that the information is expressed by four nucleic acids, i.e., adenine (A), cytosine (C), guanine (G), and thymine (T) [59]. The key factors of DNA for encryption are encoding and decoding rules, and algebraic operations for DNA sequences. Like 0 and 1 are complementary pairs in binary, 00 (0) and 11 (3), and 01 (1) and 10 (2) are also complementary pairs in DNA computing. Although there are 4!=24 combinations in total for DNA encoding, there are only 8 kinds of DNA bases are capable of meeting the DNA complementary rules, as listed in Table 1. With the encoding rule, an 8-bit pixel in grayscale image can be expressed by 4 letters. For example, following Rule 5 and Rule 8 in Table 1, the decimal gray-level 156 (’10011100’ in binary) can be transformed into a 4-letter DNA sequence ’TAGC’ orand ’ATCG’, respectively. It can be seen that for a fixed binary sequence, different rules lead to totally different DNA sequences.

In image encryption, several algebraic operations, such as addition (++), subtraction (--) and exclusive OR (XOR, ⊗⊗), as listed in Table 2, Table 3 and Table 4, can be used to change the values of nucleic acids [2].

### 2.4. Latin Square

A Latin square of order *N* is an N×N matrix which includes a set *S* with *N* different symbol elements, and each symbol shows only once in each row and each column [53]. For instance, *L* is a Latin square of order *N*, *i* and *j* represent the row and column index of an element in *L* respectively, and $S_{k}$ is the k-th element in set *S*. We can draw a formula as follows:

(2) $$f\left(i,j,k\right)=\left\{\begin{array}{c} 1,L\left(i,j\right)=S_{k}\\ 0,otherwise \end{array}\right.$$

**Figure 2:**
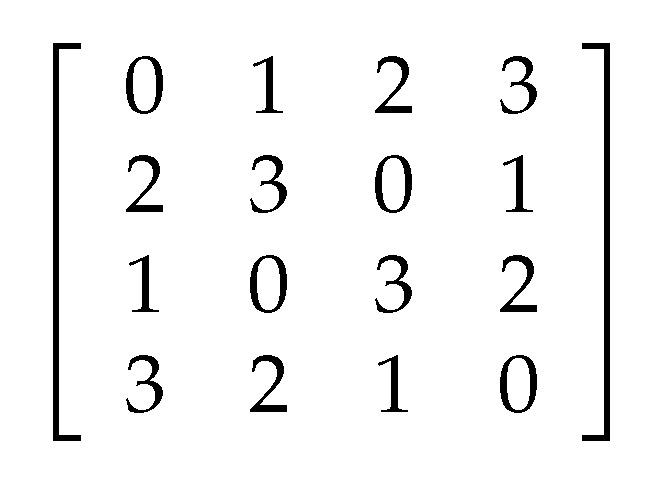
An example of Latin square of order 4.

Given S={0,1,⋯,N−1}, Figure 2 shows an example of Latin square of order 4.

## 3. The Proposed Image Encryption Approach

### 3.1. Hyperchaotic Sequence Generation

In this paper, we used the 5D hyperchaotic system described in Section 2.1 to generate the hyperchaotic sequence for encryption. Specifically, the generating process has three steps:

Step 1: The sequences generated by the first $N_{0}$ iterations are discarded to eliminate the adverse effects.
Step 2: The 5D hyperchaotic system continues to iterate to generate sequences long enough for image encryption. In the j-th iteration, we can obtain five state values denoted as $s^{j}=\left\{x_{1}^{j},x_{2}^{j},\cdots ,x_{5}^{j}\right\}$.
Step 3: When the iteration completes, a hyperchaotic sequence *S* can be obtained by contacting all the $s^{j}\left(j=1,2,\cdots ,N\right)$ as
  (3) $$\begin{array}{cc} S=\{s^{1},s^{2},\cdots ,s^{N}\} & =\{x_{1}^{1},x_{2}^{1},\cdots ,x_{5}^{1},\cdots ,x_{1}^{N},x_{2}^{N},\cdots ,x_{5}^{N}\}\\ & =\{s_{1},s_{2},s_{3},\cdots ,s_{5N-2},s_{5N-1},s_{5N}\}. \end{array}$$

The real value sequence *S* is further mapped to an integral sequence as Equation (4):

(4) $$k_{i}=mod\left(\left\lfloor mod\left(\left(\left|k_{i}\right|-\left\lfloor \left|k_{i}\right|\right\rfloor \right)\times 10^{15}\right),10^{8})\right\rfloor ,256\right),$$

where mod, $\left|\cdot \right|$ and $\left\lfloor \cdot \right\rfloor$ denote the operations of modulo, absolute value, and flooring, respectively [2,6].

### 3.2. Dynamic Filtering

**Figure 3:**
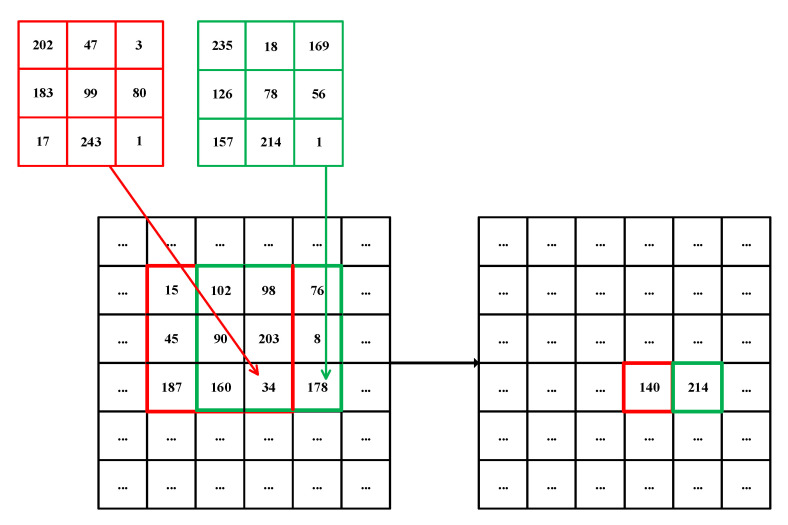
An example of dynamic filtering.

The modified filtering can be applied to image encryption, according to the very recent work BSIF by Hua and Zhou [51]. However, the BSIF does convolution on each pixel in an image with a fixed kernel generated from a random sequence. Li et al. used a 1×3 or 3×1 variable kernel to convolute each pixel in an image, that is to say, the kernels associated with each pixel for convolution are different, so-called dynamic filtering [6]. The experimental results demonstrated the effectiveness of dynamic filtering. A reasonable assumption is that a dynamic kernel with larger size (e.g., 3×3 or 5×5) will lead to better encryption. An example of dynamic filtering with two 3×3 filters is shown in Figure 3, where the 3×3 red kernel and the 3×3 blue kernel are conducted on the pixels of 34 and 178 in the plain image, and the results of dynamic filtering will be 140 and 214 in the cipher image, respectively. We can see that with dynamic filtering, the values of pixels in the plain image are changed, and this procedure can be reversible [51]. Therefore, we can use this operation for diffusion.

### 3.3. Image to Cubes

Since 3D Latin cube transformation can be conducted on cubes only, the image for encryption must be reshaped to one or several cubes. The pseudocode of such transformation algorithm (I2C) can be described as below.

Step 1: Given an image with size h×w×d, where h,w, and *d* represent the height, width, and depth, respectively, calculate the number of the pixels N=h×w×d.
Step 2: Let $L=\sqrt[{3}]{N}$, if *L* is an integer, jump to Step 3, else jump to Step 4.
Step 3: Get a cube with size L×L×L, return.
Step 4: Define $K=2^{n},n\in N$, find the biggest *K* that meets K≤L; then we get a cube with size K×K×K.
Step 5: Update $N=N-K^{3}$, if N=0, return; else jump to Step 2.

For instance, a DNA-level image with size 512×512×4 can be transformed into 4 cubes with size 64×64×64, while a DNA-level image with size 256×256×4 can be transformed into 8 32×32×32 cubes. Unlike the previous work that can only encrypts images of specified sizes [56], the proposed DFDLC can handle images of any sizes with such transformation.

Accordingly, one or several cubes can be merged into a plain image with the reverse procedure of the I2C.

### 3.4. 3D Latin Cube

**Figure 4:**
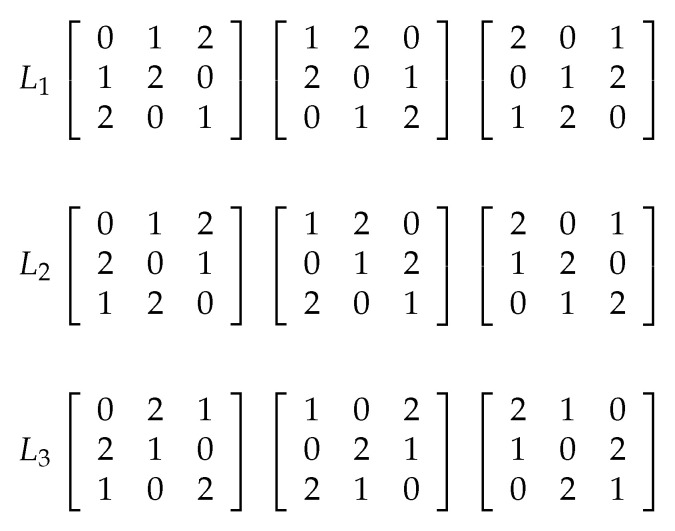
Three examples of Latin cube of order 3.

**Figure 5:**
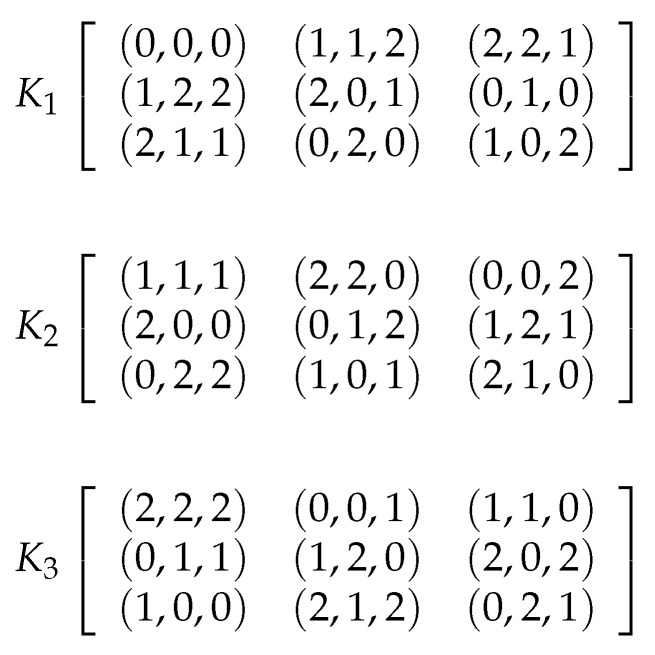
A new cube *K* constructed by $L_{1}$, $L_{2}$, $L_{3}$. $K_{1}$, $K_{2}$ and $K_{3}$ are the 1st, 2nd and 3rd squares of *K* respectively.

Latin cube is a generalized version of the Latin square. A Latin cube of order *N* is an N×N×N cube which includes a set *S* with *N* different symbol elements, and each symbol occurs only once in each row, each column, and each file [56]. Given a chaotic sequence $x=\{x_{0},x_{1},\cdots ,x_{q^{n}-1}\}$ (*q* is a prime and $q^{n}$ is the order of the Latin cubes to generate), we can sort the sequence by ascending to get an index sequence $y=\{y_{0},y_{1},\cdots ,y_{q^{n}-1}\}$ and then construct a finite field $F_{q^{n}}$ on *y* via redefining “+” and “×”. With three distinct nonzero elements $p_{1},p_{2}$ and $p_{3}$ in $F_{q^{n}}$, the element of $L_{t}\left(i,j,s\right)$ can be obtained by Equation (5):

(5) $$L_{t}\left(i,j,s\right)=y_{s}+p_{t}\times y_{j}+p_{t}^{2}\times y_{i},$$

where t={1,2,3} is the index of the Latin cube, and “+” and “×” are the addition and multiplication in $F_{q^{n}}$, respectively [56]. Figure 4 shows three Latin cubes of order 3 on the set S={0,1,2}, named as $L_{1}$, $L_{2}$, and $L_{3}$. When we superimpose the same position of three Latin cubes on the set *S*, if each combination occurs only once, we can say these three Latin cubes are orthogonal. For example, when we combine the three Latin cubes $L_{1}$, $L_{2}$ and $L_{3}$, each of the 27 combinations 000,001,002,⋯,222 occurs only once, so they are orthogonal. By combining $L_{1}$, $L_{2}$ and $L_{3}$, we can get a new cube *K* shown in Figure 5. Then a spatial permutation is obtained: (0,0,0)→(0,0,0),(0,1,0)→(1,1,2),(0,2,0)→(2,2,1),⋯,(2,2,2)→(0,2,1), i.e., the element in the left position is transferred to the right position. More generally, $K_{s}\left(i,j\right)=\left(L_{1}\left(i,j,s\right),L_{2}\left(i,j,s\right),L_{3}\left(i,j,s\right)\right)$, where *s* is the index of *K* (or *L*), and *i* and *j* are the indices of the row and the column, respectively.

### 3.5. DFDLC: The Proposed Image Encryption Approach with Dynamic Filtering and Latin Cubes

**Figure 6:**
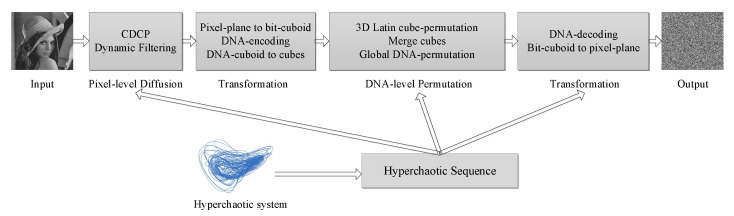
The framework of the proposed DFDLC.

The DFDLC is conducted on pixel-level diffusion and DNA-level permutation. Specifically, regarding pixel-level diffusion, we mainly apply dynamic filtering on each pixel in a plain 2D image. We also used the ciphertext diffusion in crisscross pattern (CDCP) to improve the diffusion results [60]. For DNA-level permutation, we mainly use Latin cube to change the position of each nucleic acid. In addition, a kind of global DNA permutation similar to the global bit permutation is adopted for DNA permutation [6]. The proposed DFDLC is illustrated in Figure 6. With the hyperchaotic sequence generated by the 5D chaotic system, the main steps of the DFDLC are as the following: hyperchaotic sequence generation, pixel-level diffusion, pixel-to-DNA transformation, DNA permutation, and DNA-to-pixel transformation.

The details of the DFDLC are described as follows:

Step 1: Given the keys, generate a hyperchaotic sequence with Equations (1), (3) and (4).
Step 2: Conduct CDCP with pixels of the image. This operation expands a little change in one pixel of the plain image to very large changes in a variety of pixels of the cipher image.
Step 3: Dynamic filtering on the image. For each pixel, firstly, generate a 3×3 kernel with the hyperchaotic sequence and set the right-bottom grid to 1. Secondly, do convolution with the kernel and corresponding sub-region of the image associated with the pixel. Thirdly, use the result of the convolution as the new value of the pixel in the cipher image.
Step 4: Transform the pixel image to a DNA image. For each pixel, use an encoding rule decided by the hyperchaotic sequence to encode one pixel into a string with 4 nucleic acids. The DNA encoding rule (Rule *N*) can be formulated as: N=1+mod(x,8), where *x* is a corresponding value in the hyperchaotic sequence regarding the pixel.
Step 5: Transform the DNA image into one or several cubes using I2C.
Step 6: Conduct DNA-level Latin cube permutation. For each DNA-level cube, generate a Latin cube and then change the position of each nucleic acid according to the Latin cube. In addition, the DNA XOR operation is conducted on the DNA-level cube with a generated DNA cube from the hyperchaotic sequence.
Step 7: Integrate all the DNA-level cubes into a DNA image.
Step 8: Conduct global DNA permutation as described in [2].
Step 9: Decode the DNA image into a pixel image. For each nucleic acid, the DNA encoding rule is decided as the encoding rule in Step 6. The pixel image is the cipher image.

The proposed DFDLC consists of five stages: hyperchaotic sequence generation (Step 1), pixel-level diffusion (Step 2-3), a transformation from a plane image to cubes (Step 4-5), DNA-level Latin cube permutation (Step 6-8) and a transformation from cubes to a plane image (Step 9). The keys of the DFDLC are Step 3 and Step 6, i.e., pixel-level diffusion with dynamic filtering and DNA-level permutation with 3D Latin cubes, respectively. Although the main objective of Latin cubes in the DFDLC is for permuting the DNA, it also results in diffusion because the change of the position of DNA can change the corresponding value of the pixel naturally [6].

The cipher image can be easily decrypted by the inverse steps as listed above.

## 4. Experimental Results

### 4.1. Experimental Settings

To validate the performance of the proposed DFDLC, we compare it with some state-of-the-art image encryption schemes, such as the image encryption with a fractional-order hyperchaotic system and DNA computing (FOHCDNA) [2], the hyperchaotic and DNA sequence-based method (HC-DNA) [61], CDCP [60], BSIF [51] and DFBC [6]. We set the parameters for the DFDLC as following. For the 5D hyperchaotic system, we set ($x_{1}^{0},x_{2}^{0},x_{3}^{0},x_{4}^{0},x_{5}^{0})=\left(1.618,3.14,2.718,4.6692,0.618\right)\times 10^{-2}$ and 1000 as the initial values and the preiterating times, respectively. For the compared methods, we use the parameters as set by the corresponding references.

We used ten publicly accessed images for validating the proposed DFDLC, and the details of the images are listed in Table 5.

The experiments were conducted using MATLAB 2016b (MathWorks, Natick, MA, USA) on a 64-bit Windows 7 Ultimate (Microsoft, Redmond, WA, USA) with 32 GB memory and a 3.6 GHz I7 CPU.

### 4.2. Security Key Analysis

A feasible image encryption algorithm should have a large enough key space and extreme sensitivity to the key to resist brute force attacks. In this subsection, we will analyze the key space and the sensitivity of the security key.

#### 4.2.1. Key Space

The key space is the set of all possible security keys that can be used in a system of image encryption. It was reported that the size of a key space larger than $2^{100}$ can provide enough security [62]. Basically, the 5 initial values of the 5D hyperchaotic systems, i.e., $\left(x_{1}^{0},x_{2}^{0},x_{3}^{0},x_{4}^{0},x_{5}^{0}\right)$ for Equation (1), can be constructed as the security keys. If each initial value has the same precision of $10^{-15}$, the DFDLC has a key space with size of $10^{15*5}=10^{75}\approx 2^{249}$, which is much larger than $2^{100}$. Therefore, the DFDLC can resist all types of brute-force attacks from current computers. Besides, the distinct nonzero elements in the finite filed for Latin cubes can be used as security keys to improve the key space.

#### 4.2.2. Sensitivity to Security Key

An ideal image encryption approach should be sensitive enough to the security key, that is to say, a very little change in the security keys will lead to a completely different decrypted image.

**Figure 7:**
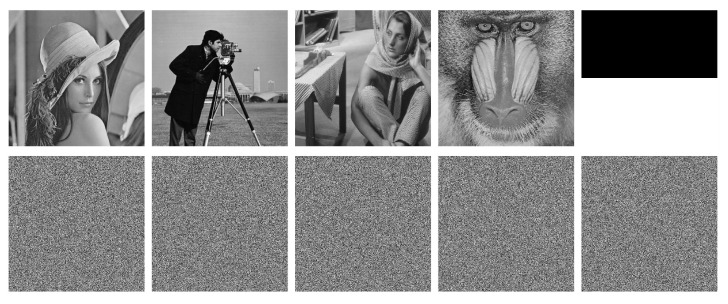
Decrypted images of Lena, Cameraman, Barbara, Mandril and Bw with security keys $g_{1}$ and $g_{2}$. The first and the second row is with $g_{1}$ and $g_{2}$, respectively.

We use two groups of slightly different keys to validate the sensitivity to the security keys of the proposed DFDLC. The first group keys are the initial values of the hyperchaotic system, i.e., $g_{1}=\left(x_{1}^{0},x_{2}^{0},x_{3}^{0},x_{4}^{0},x_{5}^{0}\right)=\left(1.618,3.14,2.718,4.6692,0.618\right)\times 10^{-2}$, while the second groups are almost the same as the first group except $x_{1}^{0}=0.0168+10^{-15}$, i.e., $g_{2}=\left(x_{1}^{0}+10^{-13},x_{2}^{0},x_{3}^{0},x_{4}^{0},x_{5}^{0}\right)=\left(1.618+10^{-13},3.14,2.718,4.6692,0.618\right)\times 10^{-2}$. We apply $g_{1}$ and $g_{2}$ to decrypt the first five images in Table 5, and the results are shown in Figure 7. It is clear that even the security keys are changed very little such as $10^{-15}$, the cipher images cannot be recovered correctly, demonstrating the high sensitivity to security keys of the proposed DFDLC [6].

### 4.3. Statistical Analysis

Statistical analysis, including histogram analysis, entropy analysis, and correlation analysis are essential for a cryptosystem. An ideal image encryption algorithm should have the ability to resist kinds of statistical attacks.

#### 4.3.1. Histogram Analysis

**Figure 8:**
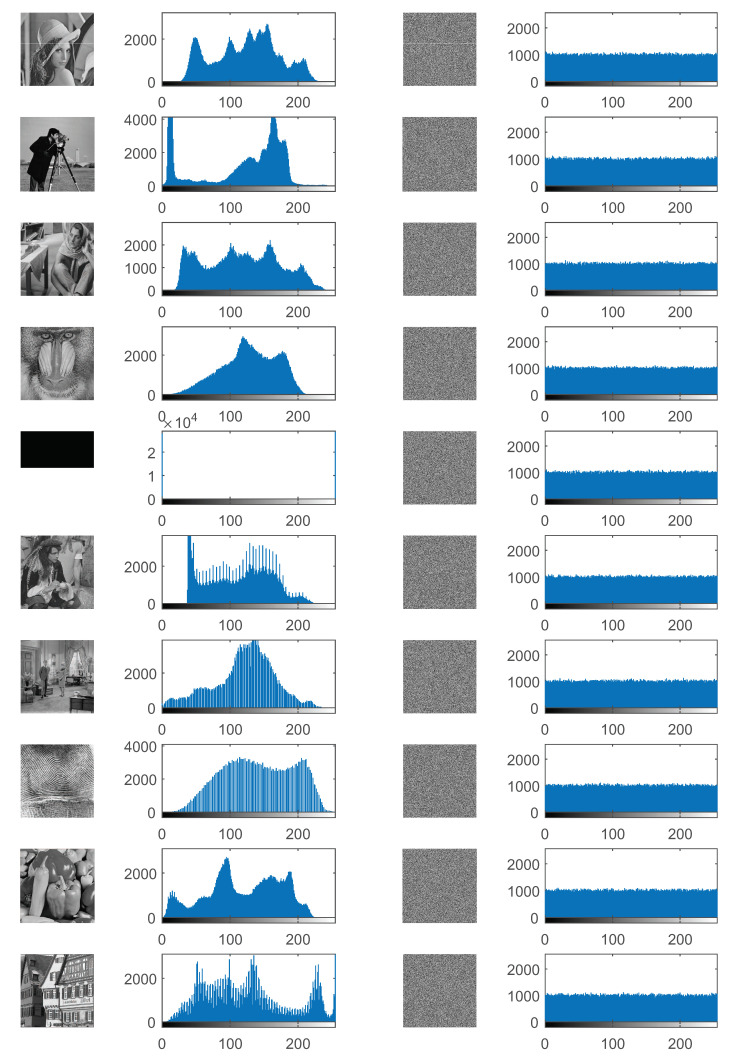
Histograms of the plain images and their corresponding cipher images. The first and the second columns are the plain images and their corresponding histograms, respectively. The third and the fourth columns are the cipher images and their corresponding histograms, respectively.

Histogram describes the distribution of pixels for an image. The histogram of a natural image usually shows an irregular (unevenly distributed) shape. A good image encryption approach should change the irregular shape of a plain image as evenly distributed as possible, leading to a completely random-like cipher image. Regarding evaluating the image encryption approach with histogram, the more uniform the histogram is, the better the encryption approach is [2]. The histograms of the plain images and the corresponding cipher images are shown in Figure 8.

It can be seen that the histograms of the plain images except Bw look like mountains, including peaks and valleys. However, the histograms of their corresponding cipher images are so flat that they are very close to uniform distributions. It is worth pointing out that regarding the image Bw, it has only two values of grayscale level, i.e., 0 and 255, and its histogram looks like two needles. However, the histogram of its cipher image is still very uniform similar to histograms of other cipher images. Although the plain images are very different, the histograms of their corresponding cipher images are so uniform and so close that it looks like that each grayscale level appears about 1000 times in all cipher images. This characteristic of cipher images can be easily found in the last column in Figure 8. The experiments indicate that the proposed DFDLC can obtain very uniform histograms for different types of images and hence it can resist histogram attacks very well.

#### 4.3.2. Information Entropy

Information entropy (IE), originally proposed by Shannon, is one of the key measures to quantify the degree of uncertainty (randomness) of a given system in information theory [63]. It can be applied to measure the randomness of an image encryption system. Given an 8-bit grayscale level that has $2^{8}=256$ possible pixel values, i.e., 0,1,⋯,255, the IE can be formulated as Equation (6)

(6) $$\mathrm{IE}\left(I\right)=-\sum_{i=0}^{255}p\left(I_{i}\right)log_{2}p\left(I_{i}\right),$$

where $p(I_{i})$ is the probability of the i-th gray value $I_{i}$ appears in an image *I*. For a cipher image, when each gray value $I_{i}$ appears with equal probability, i.e., $\frac{1}{256}$, the IE obtains the maximum 8. Therefore, an ideal image encryption approach should have an IE close to 8.

The IEs of the test images and corresponding cipher images with the DFDLC and the compared approaches are shown in Table 6. It can be seen that the testing natural images in this experiment have close IEs around 7, while the image of Bw has the lowest entropy 1, showing that the distribution of pixel values is irregular, as indicated by their histograms in Figure 8. It can be seen that the IEs of all cipher images are very close to the ideal value 8. Specifically, all encryption approaches except for HCDNA achieve very stable IEs, i.e., 7.9992∼7.9994, which are also very close to 8, indicating that these approaches are secure enough to resist entropy attacks. Although the IEs achieved by the HCDNA are slightly worse than those by the other approaches, they are still very close to the ideal value except that the IE of Bw by HCDNA is as low as 7.9158. Among the approaches, the BSIF obtains the highest IEs with 6 out of 10 cases, followed by DFDLC, FHDNA and DFBC, which all achieve the highest IEs 4 out of 10 times. However, the HCDNA achieves the highest IE only once. The experimental results demonstrate that the DFDLC are advantageous over or comparable to other approaches in terms of IE.

As mentioned above, the IEs reflect the randomness of the grayscale values in an image. The IEs achieved by DFDLC are very close to 8, indicating that the pixel values are distributed very uniformly, as the histograms shown in the last column in Figure 8. Therefore, the results of histograms are consistent with the analysis of IEs, confirming that the proposed DFDLC has good statistical properties in terms of image encryption.

#### 4.3.3. Correlation Analysis

Natural images usually show high correlation, that is, neighboring pixels have very close grayscale levels. When an image is permutated, the neighboring pixels will be randomly distributed in the whole image and hence the high correlation in plain image is broken. An ideal image encryption approach should decrease the correlation to zero in the cipher image. One of the popular ways to measure the correlation in images is the correlation coefficient γ defined as Equation (7) [6,64]

(7) $$\begin{array}{cc} E(x) & =\frac{1}{M}\sum_{i=1}^{M}x_{i},\\ S(x) & =\frac{1}{M}\sum_{i=1}^{M}{\left(x_{i}-E\left(x\right)\right)}^{2},\\ cov(x,y) & =\frac{1}{M}\sum_{i=1}^{M}\left(x_{i}-E\left(x\right)\right)\left(y_{i}-E\left(y\right)\right),\\ \gamma & =\frac{cov(x,y)}{\sqrt{S(x)S(y)}}, \end{array}$$

where *x* and *y* are grayscale levels of two adjacent pixels in an image, and *M* denotes the number of pairs of involved pixels, and E(x), S(x) and cov(x,y) are the expectation of *x*, the standard deviation of *x* and the covariance of *x* and *y*, respectively.

To analyze the correlation, we firstly use all the pairs of adjacent pixels from each plain image and the corresponding cipher image in the horizontal direction, the vertical direction, and the diagonal direction to compute the correlation coefficients, denoted by $\gamma_{h}$, $\gamma_{v}$ and $\gamma_{d}$, respectively. The results are shown in Table 7. We can see that the correlation coefficients of all plain images in all directions are very high, especially the $\gamma_{h}$ of the image Bw equals to the maximum value of 1. However, all the correlation coefficients of the encrypted images decrease to close to zero, showing that the high correlation in plain images is broken. Regarding the encryption approaches, each outperforms others in several cases, indicating they are comparable in terms of reducing the correlation in images. If we consider the range of the γ achieved by the approaches, we can see that the ranges by DFDLC, FHDNA, HCDNA, CDCP, BSIF and DFBC are [−0.0023,0.0030], [−0.0049, 0.0057], [−0.0032, 0.0038], [−0.0032, 0.0028], [−0.0032, 0.0034] and [−0.0029, 0.0027], respectively. Accordingly, the interval widths of γ by the approaches are 0.0053,0.0106,0.0070,0.0060,0.0066 and 0.0056. Among the interval widths, the DFDLC achieves the narrowest one, indicating that the DFDLC is the most stable approach in terms of γ.

**Figure 9:**
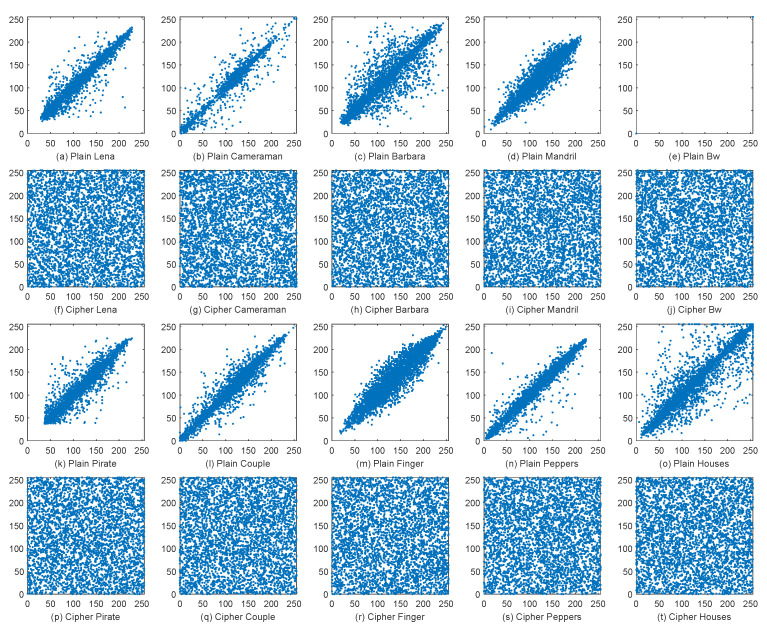
The adjacent-pixel distribution maps of the plain images and the corresponding cipher images in horizontal direction.

Then, we randomly select 4000 pairs of horizontally adjacent pixels from each plain image and its corresponding cipher image to plot the distribution maps of the grayscale levels of the adjacent pixels, as shown in Figure 9. It can be seen that the correlation of natural images is so strong that the grayscale levels of the adjacent pixels are concentrated near the diagonal line. The figure of the plain Bw is a special case because its distribution has only two possible combinations, i.e., (0,0) and (255,255). The strong correlation of all the plain images is thoroughly destroyed by the proposed DFDLC so that the grayscale levels of adjacent pixels are evenly distributed over the entire plane. It further demonstrates that the DFDLC has good performance regarding correlation.

### 4.4. Analysis of Resisting Differential Attacks

Differential attack is to study how a tiny change in a plain image can affect the corresponding cipher image. A good encryption approach should have the ability to resist differential attacks, that is to say, any small changes (even if changing a bit) in a plain image will result in a completely different cipher image. Two of the most popular indices to quantify the performance of resisting differential attacks in image encryption are the number of pixels change rate (NPCR) and the unified average changing intensity (UACI), as defined by Equations (8) and (9), respectively [65]

(8) $$\mathrm{NPCR}=\frac{1}{W\times H}\sum_{i=1}^{W}\sum_{j=1}^{H}d_{ij}\times 100\%,$$

(9) $$\mathrm{UACI}=\frac{1}{255\times W\times H}\sum_{i=1}^{W}\sum_{j=1}^{H}\left|C_{ij}^{1}-C_{ij}^{2}\right|\times 100\%,$$

where *W* and *H* denote the width and the height of the cipher images respectively, $C^{1}$ and $C^{2}$ are two cipher images, and $d_{ij}$ is defined as Equation (10)

(10) $$d_{ij}=\left\{\begin{array}{cc} 0, & C_{ij}^{1}=C_{ij}^{2},\\ 1, & C_{ij}^{1}\neq C_{ij}^{2}. \end{array}\right.$$

As far as the two indices are concerned, the NPCR focuses on the variation ratio of two cipher images whose plain images are slightly changed while the UACI defines the mean intensity of the two cipher images. Wu et al. proposed a threshold and a range for NPCR and UACI respectively to evaluate if an encryption approach can pass the differential attack test for a given specified size image at a significance level α. Specifically, for a 512×512 8-bit grayscale image, if the NPCR score is bigger than the threshold $N_{0.05}^{*}=99.5893\%$, it passes the NPCR test at α=0.05. In addition, if the UACI score falls into the interval $\left(U_{0.05}^{*l},U_{0.05}^{*u}\right)=\left(33.3730\%,33.5541\%\right)$, it is said to pass the UACI test at α=0.05 [65].

We add 1 to the value of a randomly selected pixel to compute one score of the NPCR and the UACI. The computation is repeated 10 times and then the mean, standard deviation, and times of passing the test of NPCR and UACI are reported in Table 8 and Table 9, respectively. The mean scores that pass the NPCR or the UACI tests at a significance level α=0.05 are shown in bold. One can see that both DFDLC and BSIF can pass both tests on all images in terms of the mean scores of NPCR and UACI, while CDCP and DFDC can pass most tests. In contrast, the FHDNA and the HCDNA failed the tests with all images, although the mean scores by the FHDNA are very close to $N_{0.05}^{*}$ and $\left(U_{0.05}^{*l},U_{0.05}^{*u}\right)$. If we look at the times of passing the NPCR test, both the DFDLC and the BSIF can pass the test in 99 out of 10×10=100 times and they are far superior to other methods. However, regarding times of passing the UACI test, the DFDLC is slightly worse than the BSIF, but it outperforms other methods. The experimental results demonstrate that the proposed DFDLC is capable of resisting differential attacks.

### 4.5. Discussion

The proposed DFDLC conducts encryption on pixel-level and DNA-level, with dynamic filtering for diffusion and Latin cubes for permutation. From the above analysis, we can see that the DFDLC can resist brute force attacks, statistical attacks as well as differential attacks, and the experiments have also demonstrated that DFDLC is superior or comparable to the compared state-of-the-art image encryption methods. In addition, the proposed I2C allows the DFDLC to handle images with any sizes, making it more practical.

One limitation of the DFDLC is the running time. It takes about 0.84s and 3.15s to encrypt an image of size 256×256 and 512×512 respectively in our experimental environment. The DFDLC is time consuming because the DNA operations (DNA encoding, decoding and algebraic operation) are actually operations on strings. This can be resolved by introducing lookup tables of DNA operations. Another possible way is to use GPU to accelerate DNA operations.

## 5. Conclusions

Image encryption is one of the core tasks of image security. To improve image security, in this paper, a novel image encryption algorithm that uses a 5D hyperchaotic system with 2 positive LEs, pixel-level dynamic filtering, DNA computing, and 3D Latin cubes, namely DFDLC, is proposed. The novelty of the DFDLC is introducing a new type of dynamic filtering to conduct pixel-level diffusion and permutating images with DNA-level data via Latin cubes. Extensive experiments on ten public test images have indicated that the proposed DFDLC has a large key space, is very sensitive to security keys, has good statistical characteristics, and can resist types of attacks. In the future, we will extend the proposed DFDLC in several aspects. First, we will apply trigonometric polynomials to generate the hyperchaotic sequence for the DFDLC. Second, we will try a variety of shapes of the filters for dynamic filtering. Third, we may use GPU or lookup tables to speed up the encoding and decoding of DNA and corresponding arithmetic operations. Finally, we can apply the DFDLC to color image encryption.

## Figures and Tables

**Table 1:** Encoding and decoding rules of DNA.

RULE	Rule 1	Rule 2	Rule 3	Rule 4	Rule 5	Rule 6	Rule 7	Rule 8
00	A	T	T	A	C	G	C	G
01	C	G	C	G	A	A	T	T
10	G	C	G	C	T	T	A	A
11	T	A	A	T	G	C	G	C

**Table 2:** Addition (++) operation.

++	A	C	G	T
A	C	A	T	G
C	A	C	G	T
G	T	G	C	A
T	G	T	A	C

**Table 3:** Subtraction (--) operation.

--	A	C	G	T
A	C	G	T	A
C	A	C	G	T
G	T	A	C	G
T	G	T	A	C

**Table 4:** XOR (⊗⊗) operation.

⊗⊗	A	C	G	T
A	A	C	G	T
C	C	A	T	G
G	G	T	A	C
T	T	G	C	A

**Table 5:** Testing images.

Image	Size (h×w)	Image	Size (h×w)
Lena	512×512	Cameraman	512×512
Barbara	512×512	Mandril	512×512
Bw	512×512	Pirate	512×512
Couple	512×512	Finger	512×512
Peppers	512×512	Houses	512×512

**Table 6:** The IEs of the testing images.

Image	Input	Cipher Images					
		DFDLC	FHDNA [2]	HCDNA [61]	CDCP [60]	IC-BSIF [51]	DFBC [6]
Lena	7.4455	7.9993	7.9993	**7.9994**	7.9993	**7.9994**	**7.9994**
Cameraman	7.0480	7.9992	**7.9993**	7.9981	**7.9993**	**7.9993**	7.9992
Barbara	7.6321	7.9993	**7.9994**	7.9993	7.9992	7.9993	7.9993
Mandril	7.2925	**7.9994**	7.9992	7.9992	7.9993	7.9993	7.9993
Bw	1.0000	**7.9993**	7.9992	7.9158	7.9992	**7.9993**	**7.9993**
Pirate	7.2367	**7.9994**	7.9993	7.9988	7.9993	**7.9994**	7.9993
Couple	7.0572	**7.9993**	7.9992	7.9992	**7.9993**	7.9992	**7.9993**
Finger	6.7279	7.9993	**7.9994**	7.9990	7.9992	**7.9994**	7.9993
Peppers	7.5925	7.9993	**7.9994**	7.9991	7.9993	7.9993	**7.9994**
Houses	7.6548	7.9992	7.9993	7.9993	**7.9994**	**7.9994**	7.9993

**Table 7:** The correlation coefficients γ of the testing images.

Image	γ	Input	Cipher Images					
			DFDLC	FHDNA [2]	HCDNA [61]	CDCP [60]	IC-BSIF [51]	DFBC [6]
Lena	$\gamma_{h}$	0.9691	0.0023	**0.0000**	−0.0015	−0.0004	−0.0032	0.0002
	$\gamma_{v}$	0.9841	**0.0009**	−0.0022	−0.0020	0.0028	0.0013	0.0010
	$\gamma_{d}$	0.9639	0.0008	**0.0004**	0.0024	0.0016	−0.0009	0.0006
Cameraman	$\gamma_{h}$	0.9830	0.0011	0.0013	0.0004	**−0.0001**	−0.0015	−0.0008
	$\gamma_{v}$	0.9887	0.0009	0.0033	**0.0003**	0.0019	0.0010	−0.0013
	$\gamma_{d}$	0.9746	−0.0002	**−0.0000**	−0.0013	0.0010	−0.0012	−0.0002
Barbara	$\gamma_{h}$	0.8940	−0.0003	−0.0022	0.0010	−0.0026	**−0.0002**	0.0027
	$\gamma_{v}$	0.9572	0.0030	**−0.0002**	0.0004	0.0006	−0.0004	−0.0029
	$\gamma_{d}$	0.8942	−0.0029	**−0.0000**	−0.0009	0.0005	0.0010	−0.0005
Mandril	$\gamma_{h}$	0.9322	0.0022	0.0016	−0.0007	0.0012	0.0026	**−0.0006**
	$\gamma_{v}$	0.9100	0.0005	0.0035	**−0.0001**	0.0009	**−0.0001**	−0.0018
	$\gamma_{d}$	0.8647	−0.0023	−0.0025	−0.0017	−0.0004	**0.0001**	0.0016
Bw	$\gamma_{h}$	1.0000	0.0019	0.0006	0.0004	−0.0004	0.0003	**0.0000**
	$\gamma_{v}$	0.9922	−0.0006	0.0009	0.0013	**0.0001**	−0.0005	−0.0002
	$\gamma_{d}$	0.9961	−0.0012	−0.0012	**−0.0002**	0.0005	**0.0002**	−0.0016
Pirate	$\gamma_{h}$	0.9593	**−0.0000**	0.0015	−0.0023	−0.0012	−0.0026	−0.0012
	$\gamma_{v}$	0.9675	0.0009	0.0057	**−0.0000**	−0.0008	−0.0006	0.0013
	$\gamma_{d}$	0.9432	0.0015	**0.0001**	0.0011	0.0006	0.0005	0.0005
Couple	$\gamma_{h}$	0.9451	0.0012	0.0013	0.0014	**−0.0001**	−0.0006	−0.0009
	$\gamma_{v}$	0.9514	0.0025	−0.0026	0.0008	**0.0001**	0.0023	0.0022
	$\gamma_{d}$	0.9116	0.0017	−0.0011	−0.0007	**0.0005**	−0.0008	−0.0024
Finger	$\gamma_{h}$	0.9343	**−0.0001**	0.0002	0.0007	−0.0023	0.0004	−0.0025
	$\gamma_{v}$	0.9168	**0.0002**	−0.0025	0.0029	−0.0032	−0.0009	0.0004
	$\gamma_{d}$	0.8664	0.0017	**0.0005**	−0.0022	−0.0010	0.0030	−0.0006
Peppers	$\gamma_{h}$	0.9733	0.0003	−0.0045	**0.0000**	−0.0003	−0.0031	0.0008
	$\gamma_{v}$	0.9763	−0.0010	−0.0049	−0.0005	**0.0003**	−0.0010	**−0.0003**
	$\gamma_{d}$	0.9650	0.0011	−0.0012	**−0.0005**	−0.0025	0.0017	−0.0010
Houses	$\gamma_{h}$	0.9077	0.0020	0.0006	0.0004	0.0026	**0.0001**	−0.0002
	$\gamma_{v}$	0.9173	0.0015	0.0004	−0.0032	**0.0002**	0.0017	0.0006
	$\gamma_{d}$	0.8439	0.0020	0.0021	0.0038	−0.0011	0.0034	**0.0002**
Range		[0.8439,1.000]	[−0.0023,0.0030]	[−0.0049, 0.0057]	[−0.0032, 0.0038]	[−0.0032, 0.0028]	[−0.0032, 0.0034]	[−0.0029, 0.0027]
Interval Width		0.1561	**0.0053**	0.0106	0.0070	0.0060	0.0066	0.0056

**Table 8:** The mean/standard deviation/times of passing the test of NPCR (%) of running the schemes 10 times (α=0.05).

Image	DFDLC	FHDNA [2]	HCDNA [61]	CDCP [60]	BSIF [51]	DFBC [6]
Lena	**99.6103**/0.0129/10	99.5814/0.0119/6	43.5948/16.8360/0	**99.6201**/0.2837/5	**99.6166**/0.0109/10	**99.5995**/0.0002/10
Cameraman	**99.6055**/0.0126/10	99.5795/0.0137/4	64.6306/31.1442/0	**99.6146**/0.2372/6	**99.6057**/0.0121/10	**99.6143**/0.0002/10
Barbara	**99.6171**/0.0075/10	99.5842/0.0099/8	37.8473/19.6663/0	**99.6048**/0.2136/6	**99.6165**/0.0144/10	99.5833/0.0002/10
Mandril	**99.6047**/0.0117/10	99.5774/0.0125/3	51.2024/28.3679/0	99.5697/0.2107/4	**99.6070**/0.0117/10	**99.5998**/0.0001/10
Bw	**99.6030**/0.0094/10	99.3196/0.2433/1	47.5142/15.7628/0	**99.6362**/0.2321/5	**99.6180**/0.0142/10	**99.6033**/0.0000/10
Pirate	**99.6176**/0.0133/10	99.5812/0.0127/4	35.8150/27.9995/0	**99.6403**/0.3222/7	**99.6116**/0.0116/10	99.5751/0.0002/0
Couple	**99.6089**/0.0133/10	99.5779/0.0076/5	58.1698/27.5116/0	99.5718/0.1939/3	**99.6079**/0.0112/10	99.5586/0.0001/0
Finger	**99.6097**/0.0158/10	99.5792/0.0152/4	60.3329/29.8886/0	**99.5984**/0.1420/6	**99.6171**/0.0096/10	**99.6132**/0.0002/10
Peppers	**99.6099**/0.0154/9	99.5800/0.0119/5	45.6316/38.2206/0	99.5493/0.2509/4	**99.6099**/0.0150/9	**99.6166**/0.0001/10
Houses	**99.6130**/0.0126/10	99.5795/0.0083/6	63.0733/19.2267/0	**99.6039**/0.2053/7	**99.6135**/0.0119/10	**99.6151**/0.0001/10

**Table 9:** The mean/standard deviation/times of passing the test of UACI (%) of running the schemes 10 times (α=0.05).

Image	DFDLC	FHDNA [2]	HCDNA [61]	CDCP [60]	BSIF [51]	DFBC [6]
Lena	**33.4504**/0.0466/9	33.2700/0.0490/0	18.5974/9.5490/0	**33.5212**/0.0775/6	**33.4714**/0.0339/10	**33.4818**/0.0005/10
Cameraman	**33.4909**/0.0457/9	33.3010/0.0320/0	27.0047/13.9227/0	**33.4222**/0.0658/7	**33.4755**/0.0485/10	**33.4406**/0.0005/10
Barbara	**33.4451**/0.0350/10	33.2533/0.0431/0	13.6480/8.2289/0	**33.4464**/0.1075/7	**33.4722**/0.0476/9	**33.4808**/0.0007/10
Mandril	**33.4704**/0.0334/10	33.2988/0.0336/0	22.2006/14.1993/0	**33.4467**/0.0928/5	**33.4449**/0.0423/10	**33.5136**/0.0003/10
Bw	**33.4334**/0.0471/10	32.0705/1.0272/0	18.5654/6.1072/0	**33.4555**/0.1144/4	**33.4500**/0.0468/10	41.6585/0.0010/0
Pirate	**33.4736**/0.0275/10	33.3021/0.0431/1	14.8888/14.1945/0	**33.4664**/0.0766/8	**33.4644**/0.0328/10	**33.4668**/0.0003/10
Couple	**33.4282**/0.0385/9	33.2796/0.0381/0	17.8782/7.3362/0	**33.4293**/0.1011/9	**33.4632**/0.0439/10	**33.4717**/0.0003/10
Finger	**33.4311**/0.0504/8	33.2907/0.0413/0	26.0775/14.9380/0	**33.4911**/0.1004/6	**33.4856**/0.0399/9	**33.5263**/0.0006/10
Peppers	**33.4618**/0.0432/10	33.2735/0.0347/0	19.8106/18.6275/0	**33.4626**/0.0752/6	**33.4301**/0.0379/10	**33.4525**/0.0009/10
Houses	**33.4634**/0.0358/10	33.3322/0.0273/1	22.1296/7.4892/0	**33.4721**/0.0467/10	**33.4448**/0.0343/10	**33.4545**/0.0004/10
